# The Olfactory Origins of Affective Processing: A Neurobiological Synthesis Through the Walla Emotion Model

**DOI:** 10.3390/life16010086

**Published:** 2026-01-07

**Authors:** Peter Walla

**Affiliations:** 1 Freud CanBeLab, Faculty of Psychology, Sigmund Freud Private University, Freudplatz 1, 1020 Vienna, Austria; peter.walla@sfu.ac.at; 2 Faculty of Medicine, Sigmund Freud Private University, Freudplatz 3, 1020 Vienna, Austria; 3 School of Psychology, Newcastle University, University Drive, Callaghan, NSW 2308, Australia

**Keywords:** olfaction, affection, non-conscious processing, unconscious processing, startle reflex modulation, cognition, emotion, feeling, affective processing, smelling

## Abstract

This essay provides a neurobiological and neuroanatomical analysis of how the recently published Walla Emotion Model, with its neurobiologically grounded definitions, elucidates the evolutionary origin of affective processing from the sense of olfaction. The analysis first deconstructs the model’s hierarchical framework, which distinguishes between rapid, non-conscious affective processing (neural activity coding for valence of stimuli), conscious, subjective feelings, and observable, communicative emotions. It then details the unique neuroanatomical pathway of the olfactory system, highlighting its most direct, subcortical connections to the limbic system (only two synapses) (shared subcortical network between olfaction and affection). The core argument presented is that this emotion model’s definition of affective processing as being distinct from an emotion is a direct conceptual reflection of the ancient, hardwired, and survival-oriented function of olfaction. This link is substantiated by empirical evidence from studies on sniffing behavior, startle reflex modulation, and non-conscious physiological responses, all of which provide empirical evidence for a non-conscious, non-cognitive evaluation of olfactory stimuli. First, this essay concludes that a clear distinction between affective processing, feelings, and emotions offers a coherent framework that has the potential to resolve long-standing terminological ambiguities in the affective science. Second, it also aims at providing a paradigm for understanding the foundational role of a specific sensory modality in the evolution of our most primitive and yet so evident and impactful affective responses serving the adaptation of produced behavior in humans. Finally, some ideas for broader implications are mentioned.

## 1. Introduction

### 1.1. The Walla Emotion Model—A New Taxonomy for Affective Science

In the field of “emotion” research, a pervasive problem has been the ambiguous and interchangeable use of terms such as “affection,” “feeling,” and “emotion.” This terminological imprecision has hindered rigorous scientific inquiry and obscured the neurobiological distinctions between different components that work together but are nevertheless separate phenomena. The Walla Emotion Model (also reported as the ESCAPE (Emotions Convey Affective Processing Effects) Model) was developed to address this very issue by proposing a new and more neurobiologically grounded taxonomy related to the terms affective processing, feeling, and emotion [1]. The most radical aspect of this model is that it separates these three phenomena, each one serving a unique function, from basic survival to subjective experience and to complex social communication. Perhaps the most obvious difference in comparison to other models is that emotions are understood as behavioral output resulting from non-conscious affective processing, which in turn is defined as neural activity coding for valence of external and internal stimuli (i.e., evaluating stimuli). Most other models use the term emotion as an umbrella term including various phenomena. Mostly, an emotion is understood as an internal state, not as a signal to the external world. By giving an emotion the character of a signal, affective processing becomes the most crucial phenomenon in terms of forming the basis for a feeling to occur (and also emotions).

Affective processing serves the brain to make decisions on how to adapt behavior with information related to “how” a stimulus is [2]. It is thus the key to modulating an action tendency with respect to valence (how good or bad is a stimulus?). It is characterized as a rapid, non-conscious neural evaluation of a stimulus based on its valence (pleasantness or unpleasantness) and arousal (intensity). Level of arousal represents the magnitude of valence, which finally feeds into motivation-circuits where decisions are made regarding executing behavior, or not. Affective processing is believed to be an “evolutionary ancient mechanism” that occurs in older, subcortical brain regions, particularly within the limbic system, to guide immediate behavioral tendencies such as approach or avoidance [3]. It represents the brain’s instinctual, non-conscious assessment of whether something is “good or bad, dangerous or safe,” long before an individual becomes consciously aware of this evaluation (although it is connected with cognitive networks, it is initially independent from cognition that happens largely on cortical, not subcortical, levels). Crucially, according to the Walla model, it is not an emotion that offers an action tendency (most other models would say so); it is rather affective processing that delivers respective output. One next level in the hierarchy is feelings. This level might be seen parallel to the generation of emotions, or at least independent from emotions (when understood as communicative behaviors). Feelings are the conscious, subjective experiences that emerge from suprathreshold affective processing that leads to the release of chemicals in the brain through hypothalamic (and pituitary gland) involvement. As explained further below, there are strong connections between neural structures that process evaluation-related information and the hypothalamus, which together with its associated pituitary gland controls the release of chemicals. Feelings are the felt (perceived, or consciously interpreted) internal bodily responses (i.e., the released chemicals and their effects) that we become aware of (because humans are capable of consciousness), such as a racing heart, a sense of warmth, or a knot in the stomach (felt bodily responses). A crucial distinction made within the Walla model is that, for instance, concepts like “fear,” “love,” or “hate” are not emotions but feelings. These are internal, conscious states that are distinct from how they might be outwardly expressed through emotions, which becomes particularly obvious when communicative expressions are intentionally generated, like a fake smile (i.e., voluntary emotion-behavior). While emotion-behavior primarily occurs in response to affect-laden stimulation, it can, at least in humans, also be intentionally generated (mainly in social settings). Finally, rather than being internally felt states, emotions are defined as the “behavioral outputs or expressions that communicate feelings to others” [1]. These are observable actions like a “scared face,” a gasp, or a specific type of body language or body posture (perhaps even singing a song). According to this framework, an emotion is a signal to the external world, not an internal state itself (also not neural information processing). For example, a person experiencing the feeling of fear would display a “scared face,” which is the emotion, in response to affective processing, which evaluates external stimuli as “dangerous”. This clear separation of internal experience from external expression allows for a more rigorous analysis of both components. It also helps for a more precise communication and even diagnosis in the realm of psychological disorders, almost all of which include some sort of “emotional problem”. Interestingly, if science does not provide us with a clear definition of what an emotion is, how can one understand what “emotional problem” means? The model followed here aims at providing a solution. For instance, borderline personality disorder (BPD) is often associated with so-called “emotional” storms triggered by minor events. Those can be explained by extremely low-threshold affective processing leading to uncontrolled emotion-behaviors (angry face, yelling, or aggressive behavior) and strong feelings (highly active affection results in heavy release of chemicals). Chronic emptiness, which is also a BPD symptom, can be explained as a disconnection between the raw neural data (i.e., affective processing) and their cognitive interpretations. Cognition cannot translate the affective neural noise into a meaningful story, which can lead to perceived emptiness. Of course this is only speculation; however, it seems useful to separate the definition for emotion from the rest, rather than summarizing everything under the label of “emotional storm”. Even self-harm in BPD can be understood as a high-intensity emotion-behavior. By causing physical pain, a person creates a massive, clear, and “honest” sensory input. This forced input briefly synchronizes non-conscious affection with the conscious interpretation, providing a momentary sense of integration and relief from the “polluted” cognitive chaos.

The clear distinction and separation proposed by this model aligns with an evolutionary perspective, where rapid, survival-oriented evaluations (affective processing) would have necessarily evolved first, preceding the development of more complex conscious experiences (feelings) and, also, social communication (emotions). Affective processing thus forms the very basis for decision making, even still in humans. Under the assumption that affective processing is a non-conscious process, it seems obvious that it cannot be measured via self-report. The only aspect that can be measured via questionnaires is a cognitively biased (if not polluted) perceptual interpretation of what goes on in terms of subcortical affective processing. This inherent limitation of verbal surveys is a central challenge that the model addresses, mandating the use of objective physiological measures such as electroencephalography (EEG) and startle reflex modulation (SRM) to capture these initial, non-conscious neural responses. The need for these objective measures is a direct, logical consequence of the model’s core definitions and its aim to capture neural activity that is inaccessible to cognitive processes like language. Research has indeed shown that physiological measures can reveal affective responses to stimuli that are not verbally reported, thus validating this methodological approach [4,5]. This model also has the potential to solve various problems that exist in the new field of affective computing [6]. Most importantly, as mentioned above and outlined in more detail below, it is this model’s emphasis on affective processing that makes it a good candidate to support the idea that affection has its origin in olfaction. Here are the shortest possible summary and clear definitions of the model’s core components:

• Affective Processing: Neural signals coding for valence (non-conscious);
• Feeling: Perception of bodily responses (released chemicals) to affective processing;
• Emotion: Observable, communicative expressive behavior (e.g., facial expressions).

**Figure 1:**
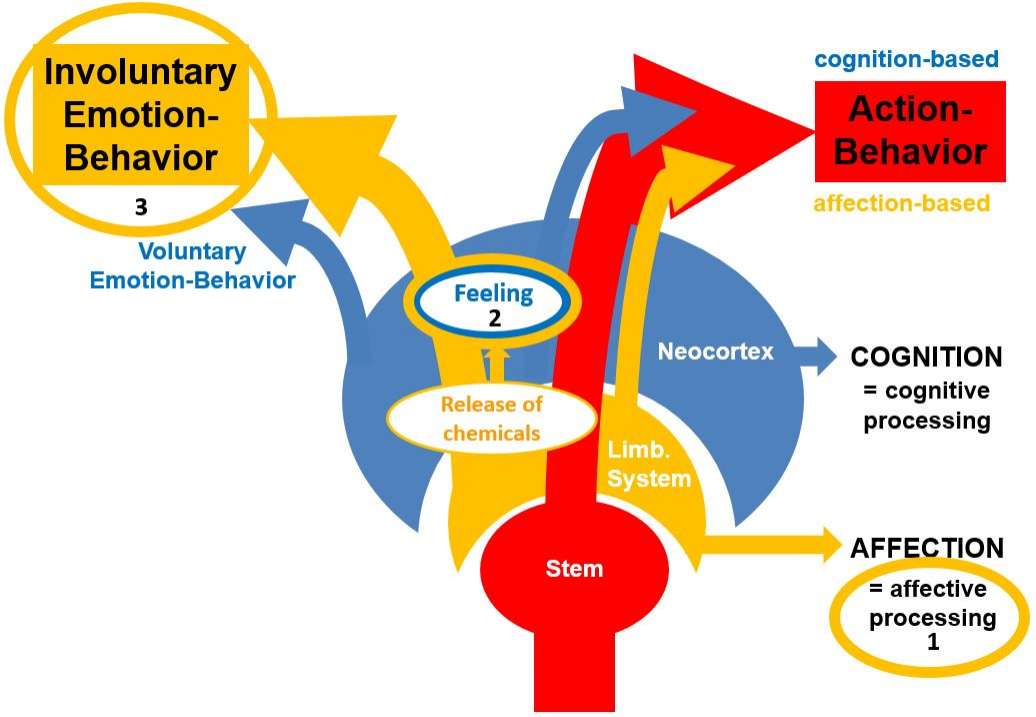
Visualization of the Walla Emotion Model (or the ESCAPE Model (Emotions Convey Affective Processing Effects)). In summary, (1) affective processing guides behavior by providing evaluative information to support decision making for the production of action-behavior. (2) Suprathreshold affective processing performed by the limbic system leads to chemicals being released under the control of hypothalamic involvement (neurotransmitters and hormones). This leads to feelings by an organism that is capable of conscious experience. (3) Involuntary emotion-behavior is produced to communicate a felt state to conspecifics. Finally, at least humans are capable of displaying voluntary emotion-behavior (mainly in social settings) (taken from Walla et al. [1]).

The following Figure 1 is meant to provide a respective visualization of the model’s core concepts including a distinction between action-behavior and emotion-behavior.

### 1.2. The Great Divide: Walla’s Model vs. Traditional Theories

To see the value of the Walla model’s concepts in order to support the notion that affective processing has its origins in olfaction, it is helpful to compare it with other foundational and established psychological theories. By no means is this comparison meant to evaluate other emotion models. The sole purpose is to try to highlight the aspects of this model’s definitions in the current context of connecting affection with olfaction. One prominent contrast is with Discrete Emotion Theory, often associated with Paul Ekman’s work on six basic emotions (anger, disgust, fear, happiness, sadness, and surprise) [7,8]. This theory posits that each emotion is a unique, discrete state with a corresponding physiological and behavioral profile. Another approach is the dimensional model [9], which suggests that emotions exist on a continuous spectrum, most commonly defined by dimensions like valence (positive/negative) and arousal (high/low intensity). The model followed here provides an important separation between the internal, subjective experience (i.e., a feeling) from the external, communicative behavior (i.e., an emotion), while ascribing the most dominant function to affective processing.

The model also diverges from Cognitive Appraisal Theories, which argue that emotions arise from an individual’s cognitive interpretation of a situation [10,11,12,13,14]. They place the emphasis on higher-level thought processes. The Walla model, by contrast, emphasizes that affective processing initially occurs non-consciously and precedes higher-level cognition leading to feelings and emotion-behavior primarily independent from cognition (as shown in phenomena such as unseen fear [15]). However, cognition can generate voluntary emotion-behavior such as fake expressions to control social interaction. In principle though, it separates clearly and strictly cognition from affection, while of course understanding that both processing qualities do interact and can influence each other.

Further, Barrett’s Theory of Constructed Emotion [16] argues that emotions are not pre-existing packages to be triggered. Instead, they are constructed by the brain in the moment. The brain uses past experience and a concept of emotion (like “anger” or “sadness”) to give meaning to internal bodily sensations, a state she calls “core affect” (a combination of valence and arousal). In this view, a person does not feel sadness because of an appraisal; rather, they construct an instance of sadness by using the concept of sadness to make sense of their feelings of unpleasantness and low energy. This understanding of what an emotion is also challenges traditional models, but there are still differences regarding key terms like “emotion” and “feeling”. Barrett’s theory sees emotions as constructed experiences and, for instance, the feeling of fear is the brain making sense of a high-arousal, unpleasant sensation by using the concept of “fear.” The Walla model agrees with the idea that the feeling of fear is the brain making sense of a high-arousal, unpleasant sensation, while calling the sensation “affective processing” and understanding the “feeling” as the perception of released chemicals in response to hypothalamic involvement.

In comparison to evolutionary theories of emotion, such as those proposed by Charles Darwin [17], this model provides a neurobiologically specific account that uses his concept of “adaptation”, or, in the current context, better defined as “decision making support”, to create a tight link between affection and olfaction. While Darwin’s work already identified the adaptive role of “emotions” (even though defining the term emotion differently) for survival, this model now adds a critical layer by explicitly linking the evolutionary function of adaptation to a specific sensory modality (olfaction) that essentially equals what affective processing does. This perspective has potential practical implications. In clinical psychology and psychiatry, differentiating between impaired affective processing, difficulties in conscious feeling, and issues with emotion-behavior can inform the diagnosis and treatment of various mental health conditions [18]. For instance, a proposed “olfactory etiology model of affective disorders” suggests that an exaggerated interaction between affection and olfaction in a negative mood state can turn innocuous odors aversive, thereby fueling anxiety and depression [19].

As will now be further highlighted below, an emotion model focusing on affective processing is well suited to strengthen the hypothesis that affection originates in olfaction from an evolutionary perspective. In short (more details below), the subcortical limbic system, which performs affective processing [2] largely through the amygdalae (consisting of at least 13 subnuclei) [20], receives most direct sensory input from the nose through the sense of smell and has connections to the hypothalamus, which controls the release of chemical substances that cause feelings. Limbic structures also have connections to motor-related neural networks to generate emotion-behavior such as automatic facial expressions. In addition, the amygdalae have direct connections to the hippocampus, which is heavily involved in important memory functions.

## 2. The Phylogenetically Ancient Link: Olfaction’s Unique Neuroanatomy

To understand how affective processing could have arisen from olfaction, it is essential to first appreciate the unique evolutionary history and neuroanatomy of the sense of smell [21]. Olfaction is widely considered the “phylogenetically most ancient sense” (at least among the mostly reported five senses (olfaction, gustation, touch, seeing, and hearing)), having evolved as a primary mechanism for survival in early vertebrates and throughout the animal kingdom [22]. The sense of smell was critical for identifying food, detecting predators, and locating mates, all of which are essential for the survival of the individual and the species. The overall function in order to support survival is the evaluation of external stimuli (i.e., are they good or are they bad). The importance of this sense throughout mammalian evolution is evidenced by the relatively large olfactory bulbs of all mammals [23]. Even birds (which are no mammals, but at least vertebrates) demonstrate relatively large olfactory bulbs [24,25]. Even though the relative size of olfactory bulbs in humans is rather small compared to other brain structures, this is more a result of other brain areas having grown exponentially large during the course of evolution rather than a sign of poor olfactory capacity. In fact, the microsmatic myth regarding humans has been proved to be wrong [26]. There is much research available that highlights the excellent human capacities regarding the sense of olfaction [27,28,29,30]. Humans might be microsmatic on a conscious level (like labeling scents), but they are macrosmatic on a neural level (affection). According to Bushdid et al. (2014), humans can discriminate 1 trillion scents [29].

The key neurobiological evidence supporting the link between olfaction and affective processing is the unique anatomical pathway that olfactory signals take in the brain. Unlike all other abovementioned sensory signals—including sight, sound, and touch—which are relayed through the thalamus, a central “relay station” for higher-level cortical processing, “smell signals bypass the thalamus” before reaching cortical layers [31]. Most importantly, nerve signals from the nose travel to the olfactory bulb, which then sends them directly to deeper structures of the limbic system, the brain region strongly associated with affective processing and memory [32]. This “unique wiring” creates a singular and potent “intimacy” between the sense of smell and the affective system, including especially episodic memory-related structures [33]. The direct connections between the olfactory bulb and key limbic structures are central to this pathway. First, the amygdala receives direct projections from the olfactory bulb. This direct link is fundamental to the ability of odors to mediate “innate attraction and aversion” and explains why smells can trigger such powerful states [34]. Second, the hippocampus, critical for memory formation and retrieval [35], receives indirect olfactory signals through the entorhinal cortex [36]. This connection is the neuroanatomical basis for the powerful link between odors and memory, which research has shown to be more vivid than memories triggered by visual cues [37,38,39]. Then, the piriform cortex interprets nerve signals into a conscious perception of a smell bypassing thalamic involvement [40], while the orbitofrontal cortex (OFC) is a key area involved in the subjective evaluation of a stimulus’s pleasantness or unpleasantness [41,42]. See Table 1, which provides a list of all abovementioned structures including short functional and connection-related aspects.

At this stage, it has to be mentioned that other senses are also connected to limbic structures, allowing for phenomena such as the abovementioned unseen fear [15]. However, the sense of olfaction is unique, because its entire primary architecture is subcortical rather than being just a secondary shortcut. In addition, as a matter of fact, olfaction is the only among the five senses (smelling, seeing, hearing, tasting, and touching) that is not filtered through the thalamus before reaching cortical layers [26].

This unique olfactory–limbic pathway suggests that the sense of smell is hardwired for immediate, affectively significant evaluation without the need for cognitive interpretation (in fact, equal to affective processing).

With respect to the current essay, the amygdala represents the most important structure. It links olfaction directly with affection. There are only two synapses between an olfactory receptor and neural cell bodies in the amygdala (no other sense has such direct connections to the amygdala). For this reason, it is important to take a closer look at all its neural connections in general. First, afferent pathways to the amygdala can be divided into five groups. Most crucially in the current context are (i) fibers from the olfactory bulb and from olfactory areas of the cerebral cortex (those are without thalamic relay), but also (ii) fibers originating in the basomedial telencephalon and the hypothalamus, (iii) thalamic afferents, (iv) direct afferents from the brainstem, and (v) projections from various non-olfactory areas of the cerebral cortex [43]. Regarding efferent pathways away from the amygdala, there are also five divisions (directions). Perhaps the most important projection is (i) to the septo-preoptico-hypothalamic continuum, which allows for a tight link between visceral (internal) states and behavioral output, ensuring, for example, that changes in body temperature can trigger both autonomic responses (sweating or shivering, regulated by the hypothalamus) and behavioral changes through the so-called medial forebrain bundle (MFB), which is one of the most important pathways for coordinating motivated behavior, affection, and internal physiological states [44]. Second, (ii) there are connections to the dorsal thalamus, then (iii) to various nuclei in the brainstem, (iv) the striatum (basal ganglia), and (v) to some cortical areas [43].

**Figure 2:**
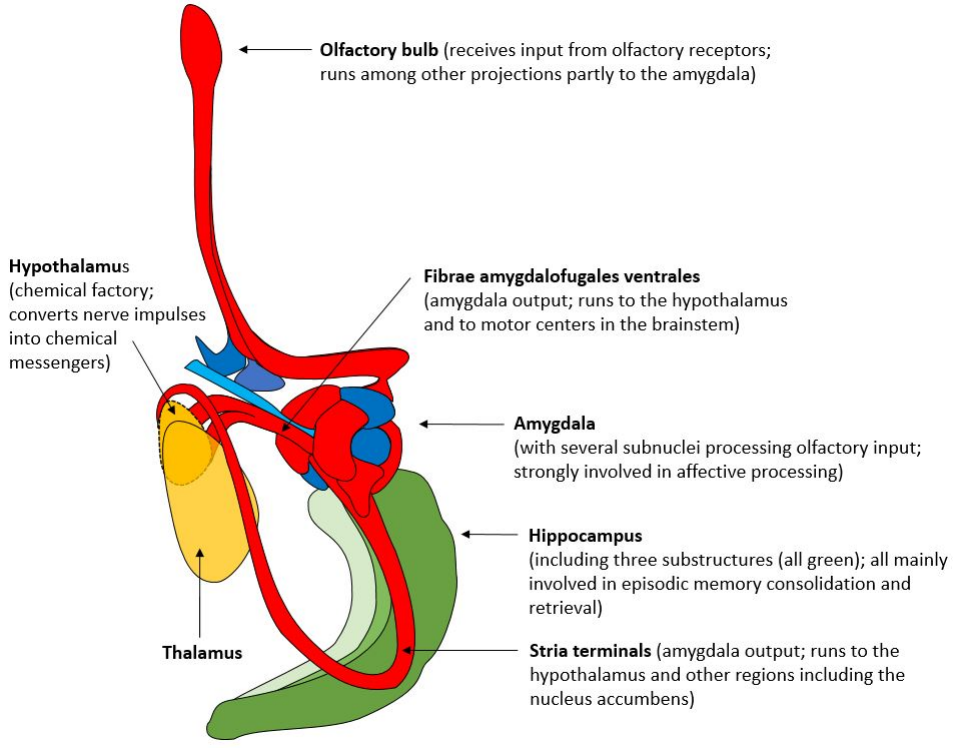
Neuroanatomical structures involved in olfactory information processing with strong overlap with affective processing (dorsal view, right hemisphere) (adapted from Nieuwenhuys et al., 2008 [43]).

Perhaps the most critical connection between feelings (as defined by Walla et al., 2025) [1] and shared affective and olfactory pathways is the hypothalamus. Price et al. (1991) [45] provide, through their anatomical tracing study, detailed evidence of olfactory projections originating from the primary olfactory areas (like the piriform cortex and amygdala) and terminating in the lateral hypothalamus. The hypothalamus can be understood as the “chemical factory” that converts nerve impulses into chemical messengers that regulate the entire body [46]. In the current context of the Walla Emotion Model, the logical neuroanatomical chain for feelings to arise (triggered via olfaction) is from the olfactory bulb to the amygdala and then to the hypothalamus. Figure 2 visualizes all those connections that are largely shared between affection and olfaction. In addition, those shared structures are also utilized to explain the rise of feelings in general, at least according to the emotion model followed here [1].

## 3. The Convergence—Explaining Affective Processing Through Olfactory Evolution

The convergence of the Walla Emotion Model’s principles, especially its fundamental emphasis on affective processing, with the unique neuroanatomy of the olfactory system provide a conceptual explanation for the origins of affective processing in the sense of olfaction [47]. Even though olfaction may simply exemplify affective processing rather than having strictly caused it during the course of evolution, the main essence that the model defines affective processing as a non-conscious, rapid, and subcortical evaluation of valence (i.e., good or bad, pleasant or unpleasant, worthy of approaching or better to be avoided) provides strong support for it. This understanding of affective processing is a precise description of the olfactory system’s unique function [21,22]. In the context of the brain’s function to produce adapted behavior, the coding for valence is the starting point. The arousal aspect adds a magnitude to valence, which results in a salience aspect. Finally, in combination with parallel cognitive information (including memory retrieval), a motivation plan is designed and executed. The primary thalamic bypass ensures that odor signals are immediately and directly routed to the limbic system, enabling a survival-critical evaluation to occur before any conscious, cognitive interpretation. This is the same in the case of affective processing. The hardwired circuitry, which mediates stereotypic attraction and avoidance behaviors, does conceptually serve as a neurobiological substrate for the Walla model’s most primitive stage of affective information processing [1].

Empirical evidence from various studies strongly supports this convergence. Research on sniffing behavior, which is the “active sampling of olfactory information”, shows that it is a direct, measurable consequence of non-conscious affective processing [47]. Studies have found that sniffing is sensitive to subtle variations in the unpleasantness of an odor, with more unpleasant odors leading to “limited… spontaneous sampling” of air [48]. This is a clear, measurable example of a non-conscious, survival-oriented motor behavior (avoidance) being guided by affective processing without the need for conscious thought. Similarly, studies using the startle reflex paradigm, which objectively measures affective state, have shown that an attractive olfactory environment leads to a reduction in the startle reflex, while an unattractive one enhances it [49]. This demonstrates how the valence of a stimulus, such as an odor, can modulate an innate reflex, further confirming the non-conscious nature of affective processing and the connection between affection and olfaction. Furthermore, neurophysiological studies using magnetoencephalography (MEG) demonstrate the close connection. Olfactory information has been shown to influence and interfere with the processing of visual stimuli (such as faces), highlighting the interaction between the affective system and olfaction [50,51] even when other senses are involved. The influence of odors on visually induced affections has also been demonstrated in other neuroimaging and behavioral studies [52].

A powerful body of evidence also highlights the dissociation between conscious perception and non-conscious affective responses. For example, in a study on appetitive Pavlovian conditioning, participants were unable to consciously distinguish between two similar odors, yet their physiological reactions—including faster inhalation and higher skin conductance responses—clearly revealed a non-conscious differentiation between the reward-associated odor and the neutral one [53]. This finding provides a direct, empirical validation of the Walla model’s central premise that affective processing, operating below the threshold of explicit perception, is distinct and measurable [54].

The evolutionary link between olfaction and affective processing also helps explain the phenomenon of feeling-charged “scent memories.” The direct projections from the olfactory bulb to the amygdala and the hippocampus ensure that an odor is not merely a cognitive cue but a fundamental part of the affective and memory encoding process itself. From the very moment of a new experience, the associated odor is routed directly to the brain’s affective and memory centers, giving the memory a powerful affective signature from its inception [55]. This is why scent-triggered memories are so “vivid,” “feeling-intense,” and often “transport you back in time,” as the feeling and memory are inextricably linked at a neurobiological level [56]. An intriguing finding related to this pathway is that simultaneous non-conscious odor processing can improve memory formation in other stimulus modalities, and this effect seems not to depend on odor valence [57]. This observation suggests that the earliest, most primitive affective processing stage is not simply a pleasant/unpleasant binary. Instead, it may be a more fundamental mechanism of “tagging” or “signifying” a stimulus’s importance for attention and memory, an ancient survival function that operates outside of the conscious pleasure/displeasure continuum. This moves the understanding of affective processing beyond a simple dimensional model and suggests an even more primitive function rooted in evolutionary preparedness.

## 4. A Comparative Analysis and Broader Implications

The connection between affection (affective processing) and olfaction could offer profound benefits to various non-neuroscience disciplines by providing an objective and evolutionary-grounded framework. In consumer neuroscience and marketing, this notion provides a potential framework for understanding how an initial, non-conscious affective response to a product’s scent [58] can influence consumer behavior and purchase decisions, often below the threshold of conscious awareness. In relation to arts, esthetics, and cultural studies, the suggested connection between olfaction and affection may provide a scientific lens for understanding the deep, often irrational power of a scent in human experience. It explains why certain smells can instantly evoke powerful, non-cognitive feelings (i.e., feelings that cannot be labeled) or transport a person back in time (scent memories). Regarding cultural significance, researchers can explore how cultural meanings and societal concepts are mapped onto basic, non-consciously evaluated affective responses (e.g., an odor that is inherently “bad” in an evolutionary sense being culturally reframed).

For forensic science and legal studies, the dissociation between conscious perception and non-conscious affective responses in general could potentially have implications for testimonial evidence and detecting deception. The focus on objective physiological measures (e.g., skin conductance; startle reflex) can perhaps be adapted to gauge an individual’s actual affective response to stimuli related to a crime, bypassing the cognitive biases inherent in verbal reports. Studies show that physiological reactions can reveal non-conscious differentiation between stimuli, even when a person cannot verbally distinguish them (not even necessarily because of lying). For instance, with startle reflex modulation, it can be shown that a psychopath responds verbally to a disgusting image (e.g., a mutilated body) with explicitly stated aversion, whereas data on non-conscious affection show significantly more positive responses compared to healthy controls [59].

In the field of Neuro-Information Systems (NeuroIS) and Technology, this link may offer superior predictive power over subjective reporting. It could be a valuable tool for understanding how a user’s non-conscious affective processing influences their behavior when interacting with technology. In the realm of affective computing, the concept of affective processing rooted in olfaction could inspire new forms of bio-feedback or affection sensing technology. In pharmacology and drug development, the olfactory system’s unique direct access to the limbic system bypassing the blood–brain barrier (BBB) could offer a potential pathway for targeted pharmacological delivery. Since odor signals project most directly to subcortical structures like the amygdala and hypothalamus, this pathway could be exploited to deliver therapeutic agents that target affective centers without depending on BBB and first-pass metabolism bypassing. Further, the proposed “olfactory etiology model of affective disorders” suggests that an exaggerated negative interaction between affection and olfaction could fuel conditions like anxiety and depression. Understanding this mechanism may help in guiding the development of pharmacological or behavioral therapies focused on modulating this specific interaction.

Finally, the recognition that olfaction is a powerful, non-conscious driver of affective evaluation highlights its importance in public spaces. Urban planners may want to move beyond visual and noise pollution to actively consider the affective impact of environmental odors. Since attraction and avoidance behaviors are hardwired into the olfactory-limbic circuitry, managing the scent landscape of cities, parks, and indoor spaces might have a positive influence on human comfort and behavior. It is emphasized that even though humans seem largely visual, which is mainly due to vision’s strong link to consciousness, olfaction is still a strong primary driver within non-conscious information processing. This might be supported by the herewith proposed tight link between olfaction and affection.

## 5. Conclusions

The Walla Emotion Model offers a framework for understanding affective neuroscience, emphasizing the foundational role of non-conscious affective processing and drawing clear lines between it, conscious feelings, and observable emotion-behaviors. The analysis presented in this essay establishes that the unique, phylogenetically ancient pathway of the olfactory system serves as a neurobiological blueprint for this model’s most primitive stage. The direct, subcortical routing of odor signals to the limbic system, bypassing the filtering of the thalamus and thus also bypassing cognition, provides a powerful functional explanation for the rapid, non-conscious evaluation of stimuli that also defines affective processing [2]. By synthesizing these two domains, the Walla model not only provides a new perspective on the long-standing terminological ambiguities in emotion research [1] but also an interesting paradigm for the neurobiological foundations of affective processing. The model’s emphasis on objective physiological measures and its ability to explain phenomena like non-conscious sniffing behavior and non-valence-dependent memory enhancement make it a valuable tool for advancing research in neuroscience, psychology, and beyond. This framework positions olfaction not merely as a sense but as a fundamental driver of our most basic, survival-critical affective responses, demonstrating that the very foundation of our complex affective and felt lives is rooted in our most ancient sense. In summary, it is here proposed that human affection could indeed have its origin in olfaction, which would be well mirrored in the recently reported new Walla Emotion Model [1]. It has to be mentioned though that this is only speculation. It is also possible that affective processing is simply exemplified by olfaction rather than originating from it. However, this essay tries to provide a coherent story where affective processing has olfactory roots, with feelings and emotion-behavior emerging later, built on that chemosensory foundation.

## Figures and Tables

**Table 1:** Summary of olfaction-related key structures.

Brain Region	Function in Olfaction	Key Connection
Olfactory Bulb	Initial processing of odor signals; creates a spatial “odor map” for chemical structure.	Receives direct input from olfactory sensory neurons (OSNs).
Piriform Cortex	Interprets nerve signals to produce the conscious perception of a smell.	Receives direct projection from the olfactory bulb.
Amygdala	An important affective processing center of the brain; also mediates innate attraction and aversion to odors.	Receives direct projection from the olfactory bulb, bypassing the thalamus.
Hippocampus	A hub for memory formation and retrieval (particularly episodic memory).	Receives signals from the amygdala and also directly from the olfactory bulb (besides indirect connections).
Orbitofrontal Cortex (OFC)	Involved in the subjective rating of an odor’s pleasantness or unpleasantness.	Receives direct projection from the olfactory bulb and primary olfactory cortex.

## Data Availability

No new data were created or analyzed in this study.
